# Associated Factors to Nonadherence to Routine Appointments after Kidney Transplantation: The ADHERE Brazil Study

**DOI:** 10.1111/ctr.70339

**Published:** 2025-10-07

**Authors:** Roberta Lopes Karlburger, João Henrique Sendrete de Pinho, Fernando Antônio Basile Colugnati, Kris Denhaerynck, José Osmar Pestana Medina, Tainá Veras de Sandes‐Freitas, Sabina De Geest, Helady Sanders‐Pinheiro, the ADHERE BRAZIL Study Team

**Affiliations:** ^1^ Renal Transplantation Unit, University Hospital, Federal University of Juiz de Fora and Núcleo Interdisciplinar De Estudos e Pesquisas Em Nefrologia Federal University of Juiz de Fora Juiz de Fora Brazil; ^2^ Institute of Nursing Science Department of Public Health University of Basel Basel Switzerland; ^3^ Academic Centre for Nursing and Midwifery Department of Public Health and Primary Care KU Leuven Leuven Belgium; ^4^ Hospital Do Rim e Hipertensão, Fundação Oswaldo Ramos, Disciplina de Nefrologia Universidade Federal de São Paulo São Paulo Brazil; ^5^ Department of Clinical Medicine Federal University of Ceara Fortaleza Brazil

**Keywords:** appointments and schedules, health services accessibility, kidney transplantation, multicenter study, patient non‐adherence

## Abstract

**Rationale:**

Nonadherence to routine outpatient appointments (NApp) post kidney transplantation (KT) is a poorly studied health behavior associated with unfavorable outcomes. In the ADHERE BRAZIL Study, we previously reported a high prevalence of this behavior (12.7%).

**Aims and Objective:**

This study aimed to identify the multilevel factors associated with NApp after KT.

**Method:**

A cross‐sectional study, subproject of the ADHERE BRAZIL Study, was performed. We studied a randomized and multi‐stage sample of 1105 patients from 20 transplant centers. Patients who missed one or more of the last five scheduled appointments were considered nonadherent. Multivariate analysis was performed using sequential logistic regression, evaluating 49 multilevel variables, according to the Ecological Model (patient, micro, meso, and macro levels).

**Results:**

Most patients were male (58.5%), with a mean age of 47.6 ± 12.6 years. The independent factors associated with NApp were, at the patient level: age (OR 0.97; 95% CI 0.96–0.99; *p* = 0.001), more than 5 years after KT (OR 2.03; 95% CI 1.38–3.00; *p* < 0.001), and nonadherence to immunosuppressives (OR 2.41; 95% CI 1.66–3.50; *p* < 0.001); at the micro level (health professionals): higher scores on the team trust scale (0–100) (OR 0.98; 95% CI 0.95–1.00; *p* < 0.079), and at the meso level (KT center): frequent (monthly) consultations (OR 1.75; 95% CI 1.10–2.77; *p* < 0.018) and scheduling difficulties (OR 1.91; 95% CI 1.16–3.17; *p* < 0.011).

**Conclusion:**

This study is the first to examine the association of health system factors with missed appointments after KT. The identified patient factors allow us to recognize patients at risk for NApp. Modifiable variables at the health professional and KT center levels suggest targets for effective interventions aiming to reduce this behavior and improve outcomes.

**Trial registration:**

ClinicalTrials.gov on 10/10/2013, NCT02066935.

## 1 Introduction

Kidney transplantation (KT) is the best treatment for patients with chronic kidney disease (CKD) in kidney failure stage because it provides better quality of life, reduction of comorbidities, and decreased health costs when compared to dialysis [1, 2, 3]. Patients undergoing KT continue to require chronic care as they need to take immunosuppressives (IMS) and manage multiple risk factors, pre‐existing comorbidities, and those that arise after KT [4, 5, 6]. Adherence to the treatment of chronic diseases encompasses the following health behaviors proposed by health professionals, are closely dependent on self‐care, and include not only the use of medications, but also the practice of physical exercise, following a diet, cessation of smoking and alcohol intake, and lab examinations and consultations [7, 8]. Among the health behaviors, nonadherence to IMS is a risk factor for worse graft survival, higher patient morbidity, and high costs, and it has been extensively studied [8, 9, 10, 11, 12, 13].

The follow‐up of the patient after KT should be performed by a multidisciplinary team, through outpatient consultations and adapted to the peculiarities of each service [4, 14, 15]. Nonadherence to routine outpatient appointments (NApp) is an easily identified health behavior yet lacks a universally accepted diagnostic definition [16]. In the few published studies in KT, the prevalence of NApp ranges from 7.6% to 46.6% [16, 17, 18, 19, 20, 21, 22]. This variability can be explained by the different definitions used regarding the percentage of absences and the duration of the analysis. An interesting definition of NApp was used in the BRIGHT (Building Research Initiative Group: Chronic Illness Management and Adherence in Transplantation) study, which classified patients as nonadherent if they missed one or more appointments (≥20%) out of the last five scheduled. This definition is valuable for research purposes but also offers reliability for application in clinical practice [23]. NApp has been correlated to worse clinical outcomes, such as late graft function, acute rejection, graft loss, and death. A strong association between NApp and nonadherence to IMS has also been described, with an additive effect on the risk of graft loss [19].

**FIGURE 1:**
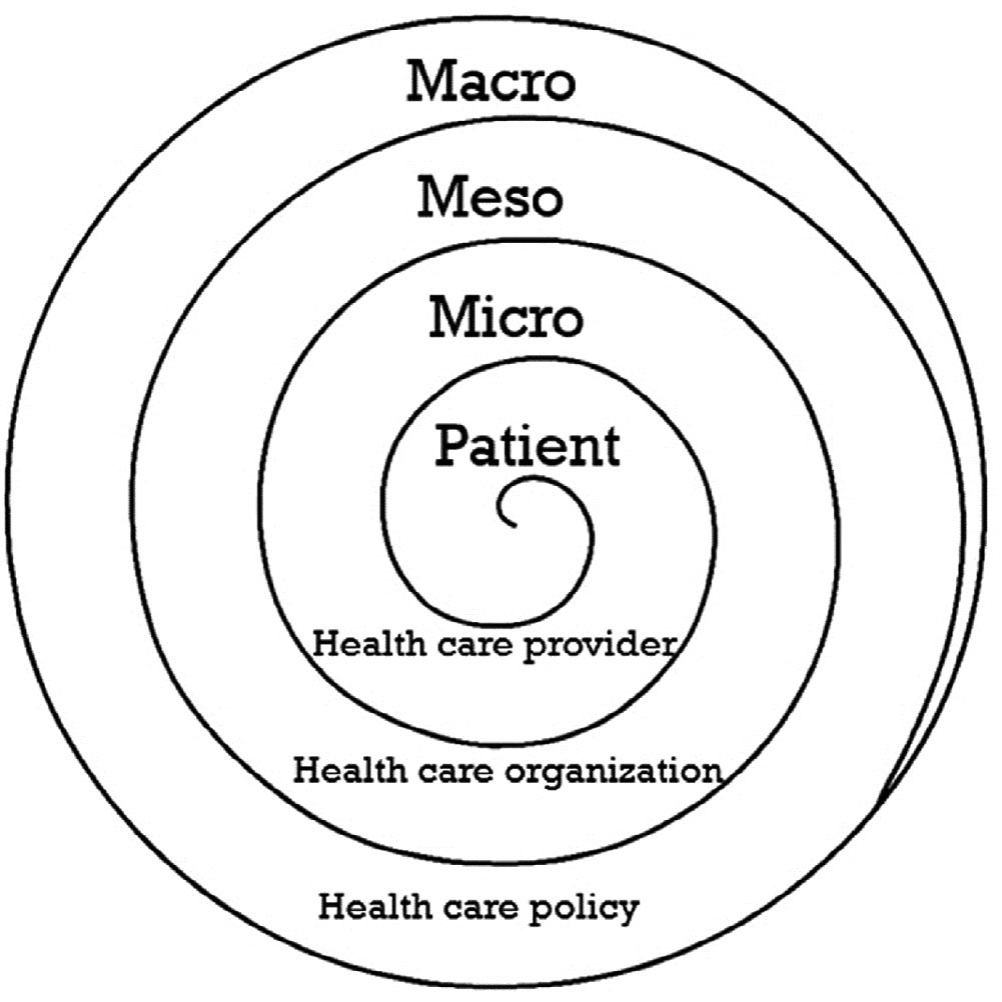
The Ecological model showing the levels that were analyzed in the study. Adapted from Berben et al. [24].

A study of correlates of health behaviors should be theory‐guided. The ecological model proposes that NApp behavior can be influenced not only by patient characteristics, but also by factors at the micro (health professional), meso (health service), and macro (health system) levels [24] (Figure 1). The few studies on potential factors associated with NApp evaluated only demographic and clinical characteristics of patients (age, sex, race, type of donor, and nonadherence to IMS) [17, 18, 19, 21, 25, 26]. Multilevel factors in view of the KT center characteristics have not yet been assessed.

The ADHERE BRAZIL study included 1105 patients from 20 Brazilian transplant centers, aiming to explore the prevalence and factors associated with nonadherence to IMS and other health behaviors of KT recipients [27]. The prevalence of NApp was at 12.7% [28]. This sub‐analysis of the ADHERE BRAZIL study aimed to identify the multilevel factors (patient, health professional, health service, health policy) associated with NApp.

## 2 Methods

The study was previously approved by the Institutional Review Board (IRB) of the University Hospital of the Federal University of Juiz de Fora (691.120/2014) and by the other IRBs of the 19 participating centers (CAAE – 27972914.1.1001.5133) [27]. The study was conducted following the ethical standards of the Helsinki Declaration of 1975, as revised in 2013.

### 2.1 Study Design

This cross‐sectional, multicenter study is a secondary data analysis of the ADHERE BRAZIL study. The ADHERE BRAZIL study was registered on the Clinical Trials (NCT02066935) website and the Open Science Framework Platform. The methodology of the study was previously described in detail [27].

### 2.2 Sample Setting

A sample size of 1130 patients was calculated to estimate the prevalence of nonadherence to IMS. This sample was calculated for population frequency studies, setting a prevalence of nonadherence to IMS of 50%, a confidence interval of 5%, using the OpenEpi program [27]. Since the highest reported prevalence of interest is 46.6%, although it was not calculated for this specific objective, the methodology used would also be adequate to estimate the prevalence of NApp [16, 17, 18, 19, 20, 21, 22, 28]. A total of 20 Brazilian transplant centers were chosen by convenience, respecting the representativeness of the Brazilian geographic regions (North/Northeast/Midwest, South/Southeast) and the activity of the transplant centers (number of transplants/year). Patients were randomly selected from among those who attended routine visits. The inclusion criteria were: age >18 years, >1 year of KT, use of blood measurable IMS, and signing an informed consent form [27].

### 2.3 Nonadherence to Routine Health Care Appointments

We used the definition adopted in the BRIGHT study [23], in which NApp was assessed through self‐reports based on the recall of the last 5 scheduled appointments. Patients who missed 1 or more of the last 5 appointments were considered nonadherent [23, 27].

### 2.4 Factors Associated With Nonadherence to Appointments

The factors associated with NApp were evaluated according to the Ecological Model (Figure 1), divided into patient level, health professional level (micro), KT service level (meso), and health system and health policy level (macro) [24]. A critical analysis of the available variables in the database of the ADHERE BRAZIL study, originally chosen as correlates to nonadherence to IMS, was performed. Forty‐nine variables were chosen for the present study, based on previous studies [16, 17, 18, 19, 20, 21, 22, 23, 24, 25, 27, 29]. A detailed description of study variables is available in Supporting Information 1. A total of 29 variables were included at the patient level, divided into socio‐demographic, clinical, treatment‐related, and behavioral factors [16, 17, 18, 19, 20, 21, 22, 23, 25, 27, 29]. At the higher health care levels, we evaluated 2 variables at the micro level (interpersonal dimension of the care, e.g., trust in KT team) [27], 16 variables at the meso level (KT practice patterns and structure, e.g., frequency of routine appointments) [23, 27], and 2 variables at the macro level (health care system characteristics, e.g., distance from the transplant center) [18, 27] (Supporting Information 1).

### 2.5 Data Collection

Data were collected and recorded by professionals trained in Research Electronic Data Capture (RedCap) from December 2015 to July 2017. Questionnaires were applied to patients and managers of the participating KT centers. Additional patient data were extracted from the medical records.

### 2.6 Statistical Analysis

For the descriptive analysis of the variables, measures of central tendency (means and medians) or measures of dispersion and proportion (standard deviations, percentages) were used. NApp was considered as a binary variable, and logistic regression analysis using generalized estimation equation (GEE) for robust inference, given the different centers, was used to evaluate the association between the variables and NApp. The variables for which an association was found in the bivariate analysis were included in the multivariate model of exploratory logistic regression, with NApp as the outcome variable. An odds ratio (OR) < 0.75 or > 1.25 and *p* ≤ 0.25 or *p* < 0.05, independent of the OR value, were used as the level of association for entry in the multivariate model. A total of 13 variables were selected for this model, nine at the patient level, one at the micro level, and three at the meso level.

In the multiple logistic regression analysis, variable entrance followed a sequential approach following the ecological model [29]. In the first analysis, the variables at the patient level were included. After this first analysis, the variables with OR < 0.75 or OR > 1.25 or *p* ≤ 0.10 were selected for the next stage. In the second analysis, the variables selected in the first part were analyzed together with the micro‐level variables. In the third and final analysis, the patient, micro‐level variables, plus the meso‐level variables, were analyzed. The variables of each level were as follows: Block 1 (patient level): age, gender, stable partner, income, type of donor, time after KT, everolimus, azathioprine, and nonadherence to IMS. These variables were added to Block 2 (micro level): trust in the team. Next, an analysis was performed, including the variables of Block 3 (meso level): transplant activity, difficulty in scheduling, and monthly frequency of the consultation, thus generating the final model. The analyses were performed using STATA (version 15, StataCorp LP, College Station, TX, USA).

## 3 Results

**FIGURE 2:**
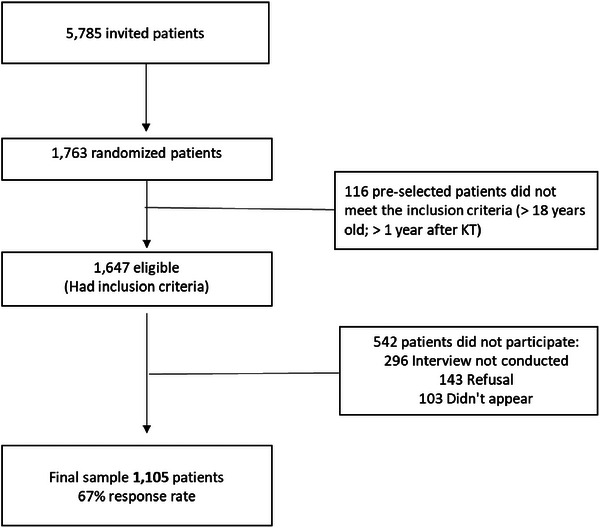
Sample flow.

Of the 1105 patients included in the study (Figure 2), 58.5% were male, 51.3% were white, with a mean age of 47.6 ± 12.6 years. The majority reported having an elementary school education (39.1%), a stable partner (60.1%), few had active work (23.3%), and a family income of 1 to 3 reference wages (52.8%). Chronic glomerulonephritis (28.6%) was the main cause of CKD, 93% were on hemodialysis before KT, post‐KT time was less than 5 years (51.13%), and 65.2% received grafts from deceased donors. Only 3.9% were smokers, most were physically inactive (69.1%), and 39.7% were classified as being nonadherent to IMS. In addition, most of them did not live in the city of the transplant center (68.9%) and did not have private health insurance (76.6%) (Table 1).

**TABLE 1:** Descriptive statistics and bivariate analysis of factors associated with nonadherence to routine outpatient appointments (NApp) in kidney transplant patients.

		Total sample	Nonadherent	Adherent	Bivariate analysis
Variable	Values/Scoring	*N*, mean ± SD, 95% CI *N* (%), 95% CI	*N*, mean ± SD *N* (%), 95% CI	*N*, mean ± SD *N* (%), 95% CI	Odds ratio (95% CI), *p* value
**Block 1: patient‐level: variables derived from empirical evidence**					
Socio‐demographic factors					
Age^a^	Years	1105, 47.6 ± 12.6	140, 43.8 ± 11.6	963, 48.7 ± 12.6	0.97 (0.96–0.99), *p *< 0.001
Sex^a^	Male	645, 58.5% (55.6–61.4)	73, 52.1% (43.9–60.3)	572, 59.4% (56.2–62.5)	0.73 (0.51–1.04), *p* = 0.085
Educational level^a^	Illiterate (0–4 years)	86, 7.8% (6.3–9.5)	9, 6.4% (3.4–11.9)	77, 8.0% (6.4–9.9)	Reference
	Elementary school (4–8 years)	431, 39.1% (36.2–41.9)	54, 38.6% (30.9–46.9)	377, 39.2% (36.1–42.3)	1.18 (0.56–2.48), *p* = 0.665
	High school (> 8 to 11 years)	421, 38.2% (35.4–41.2)	57, 40.7% (32.9–49.0)	364, 37.8% (34.8– 40.9)	1.30 (0.61–2.73), *p* = 0.494
	College (<11 years)	165, 15.0% (12.9–17.2)	20, 14.3% (9.4–21.1)	145, 15.1% (12.9– 17.5)	1.15 (0.50–2.64), *p* = 0.747
Race^a^	Caucasian	566, 51.3% (48.4–54.3)	66, 47.1% (39.0–55.4)	500, 51.9% (48.7–55.1)	—
Marital status^a^	Stable partner	662, 60.1% (57.0–62.9)	75, 53.6% (45.3–61.7)	587, 61.1% (58.0–64.1)	0.73 (0.51–1.04), *p* = 0.088
Working status^a^	No active work	846, 76.7% (74.1–79.1)	104, 74.29% (66.4–80.8)	742, 77.0% (74.3–79.6)	—
Familiar income^a^	Brazilian reference wages/month				
	1. From 0 to 1	280, 25.4% (23.0–28.0)	43, 30.7% (24.0–39.0)	237, 24.3% (0.22 – 0.27)	—
	2. > 1–3	582, 52.8% (50.0–56.0)	72, 51.4% (43.0–60.0)	510, 53.0% (0.50 – 0.56)	0.74 (0.5–1.1), *p* = 0.146
	3. >3	240, 21.8% (19.0–24.0)	25, 17.9% (12.0–25.0)	215, 22,3% (0.20 – 0.25)	0.61 (0.3–1.0), *p* = 0.073
Religion^a^	Yes	1042, 94.5% (93.0–96.0)	131, 93.6% (88.0– 97,0)	911, 94.6% (93.0–96.0)	0.84 (0.40–1.76), *p* = 0.650
**Clinical factors**					
CKD aetiology^a^	1. Chronic glomerulonephritis	315, 28.6% (26.0–31.3)	43, 30.7% (23.6–38.8)	272, 28.2% (25.5–31.2)	Reference
	2. Undetermined	315, 28.56% (25.9–31.2)	39, 27.9% (21.0–35.8)	276, 28.7% (25.9–31.6)	0.86 (0.53–1.39), *p* = 0.545
	3. Hypertensive nephropathy	222, 20.13% (17.8–22.5)	25, 17.9% (12.3–25.1)	197, 20.5% (18.0–23.1)	0.84 (0.50–1.43), *p* = 0.530
	4. Diabetic Nephropathy	96, 8.70% (7.2–10.5)	12, 8.6% (4.9–14.5)	84, 8.7% (7.1–10.7)	0.91 (0.46–1.81), *p* = 0.790
	5. Polycystic Kidney Disease	65, 5.89% (4.6–7.4)	6, 4.3% (1.9–9.2)	59, 6.1% (4.8–7.8)	0.71 (0.27–1.64), *p* = 0.382
	6. Other	90, 8.16% (6.7–10.0)	15, 10.7% (6.5–17.0)	75, 7.8% (6.2–9.7)	1.30 (0.68–2.47), *p* = 0.420
Pre‐KT treatment modality^a^	1. Hemodialysis	1026, 93.0% (91.4–94.4)	129, 92.1% (86.4–95.6)	897, 93.1% (91.4–94.6)	Reference
	2. Peritoneal dialysis	40, 3.6 % (2.7–4.9)	7, 5.0% (2.4–10.1)	33, 3.4% (2.4–4.8)	1.43 (0.61–3.33), *p* = 0.408
	3. Pre‐emptive	37, 3.3% (2.4–4.6)	4, 2.9% (1.1–7.4)	33, 3.4% (2.4–4.8)	0.82 (0.28–2.39), *p* = 0.716
Acute rejection^a^	Yes	249, 22.8% (20.4–25.4)	37, 26.8% (20.1–34.8)	212, 22.2% (19.7–24.9)	1.26 (0.83–1.9), *p* = 0.275
Creatinine	mg/dL	1103, 1.6± 0.8 (1.5–1.6)	962, 1.6 ± 0.8 (1.5–1.6)	140, 1.7 ± 1.0 (1.5–1.9)	1.15 (0.95–1.40), *p* = 0.141
Estimated GFR	CKD‐EPI equation, mL/min/1.73 m^2^	1103, 58.0±25.0 (56.5– 59.5	140, 57.0 ± 26.4 (52.6–61.4)	963, 58.2 ± 24.8 (56.6–59.7)	1.00 (0.99–1.00), *p* = 0.764
CKD categories^a^	1 (GFR >90)	122, 11.0% (9.3–13.0)	17, 12.1% (7.7–18.7)	105, 10.9% (9.1–13.0)	1.04 (0.90–1.20), *p* = 0.574
	2 (GFR 90–60)	373, 33.8% (31.0–36.6)	44, 31.4% (24.3–39.6)	329, 34.2% (31.2–37.2)	
	3a (GFR 59–45)	256, 23.2% (20.8–25.8)	29, 20.7% (14.8–28.2)	227, 23.6% (21.0–26.4)	
	3b (GFR 44–29)	206, 18.7% (16.5–21.1)	29, 20.7% (14.8–28.2)	177, 18.4% (16.0–20.9)	
	4 (GFR 30–15)	123, 11.1% (9.4–13.1)	16, 11.4% (7.1–17.8)	107, 11.1% (9.3–13.2)	
	5 (GFR <15)	23, 2.1% (1.4–3.1)	5, 3.6% (1.5–8.3)	18, 1.9% (1.2–2.9)	
Number of hospitalizations post‐KT^a^	1. None	332, 30.1% (27.5–32.9)	41, 29.3 % (22.3–37.3)	291, 30.2% (27.4–33.2)	Reference
	2. 1	279, 25.3% (22.8–27.9)	35, 25.0% (18.5–32.8)	244, 25.3% (22.3–37.3)	1.01 (0.62–1.64), *p* = 0.968
	3. 1–3	289, 26.2% (23.6–28.8)	38, 27.1% (20.4–35.1)	251, 26.1% (23.4–28.9)	1.07 (0.66–1.72), *p* = 0.783
	4. > 3	203, 18.4% (16.3–20.9)	26, 18.6% (12.9–25.9)	177, 18.4% (16.0–20.9)	1.03 (0.60–1.75), *p* = 0.921
**Treatment‐related factors**					
Time since transplantation^a^	1. Up to 5 years	564, 51.13% (48.3–54.2)	52, 37.14% (29.5–45.4)	512, 53.17% (50.0–56.3)	Reference
	2. More than 5 years	539, 48.87% (45.8–51.7)	88, 62.86% (54.5–70.4)	451, 46.83% (43.7–50.0)	1.89 (1.30–2.74), *p* = 0.001
Type of donor^a^	1. Living	384, 34.8% (32.0–37.6)	60, 42.9% (34–9–51.2)	324, 33.6% (30.7–36.7)	Reference
	2. Deceased	719, 65.2% (62.4–68.0)	80, 57.1% (48.8–65.1)	639, 66.4 % (63.3–69.3)	0.71 (0.49–1.02), *p* = 0.066
Prednisone	Yes	1046, 94.8% (93.4–96.0)	137, 97.9% (93.6–99.3)	909, 94.4% (92.7–95.7)	2.48 (0.76–8.10), *p* = 0.132
Tacrolimus	Yes	847, 76.8% (74.2–79.2)	106, 75.7% (68.0–82.1)	741, 76.9% (74.2–79.5)	0.95 (0.62–1.45), *p* = 0.814
Sirolimus	Yes	72, 6.5% (5.2–8.1)	6, 4.3% (1.9–9.2)	66, 6.8% (5.4–8.6)	0.60 (0.25–1.41), *p* = 0.242
Everolimus	Yes	138, 12.5% (10.7–14,6)	11, 7.9% (4.4–13.6)	127, 13.2% (11.2–15.5)	0.52 (0.27–1.02), *p* = 0.058
Cyclosporine	Yes	142, 12.9% (11.0–14.9)	22, 15.7% (10.6–22.7)	120, 12.5% (10.5–14.7)	1.31 (0.80–2.16), *P* 0.279
Azathioprine	Yes	153, 13.9% (11.9–16.0)	29, 20.7% (14.8–28.2)	124, 12.9% (10.9–15.1)	1.70 (1.07–2.70), *P* 0.024
Mycophenolate mofetil	Yes	83, 7.5% (6.1–9.2)	12, 8.6% (4.9–14.5)	71, 7.4% (5.9–9.2)	1.20 (0.63–2.30), *p* = 0.578
Sodium mycophenolate	Yes	698, 63.3% (60.4–66.0)	86, 61.4% (53.1–69.1)	612, 63.5% (60.4–66.5)	0.96 (66.4–1.40), *p* = 0.854
Amount of IMS doses per day	1. 1/2 times a day	858, 77.8% (75.3–80.2)	114, 81.4% (74.1–87.0)	744, 77.3% (74.5–79.8)	Reference
	2. 3 or more times	245, 22.2% (19.8–24.7)	26, 18.5 (12.9–25.9)	219, 22.7% (20.2–25.5)	0.82 (0.51–1.34), *p* = 0.447
**Behavioral factors**					
Physical activity^a^	No	762, 69.1% (66.3–71.8)	95, 67.9% (59.7–75.1)	667, 69.3% (66.3–72.1)	0.96 (0.66–1.41), *p* = 0.850
Smoking^a^	No	43, 3.9% (2.9–5.2)	5, 3.6 % (0.0149287 0.0830014	38, 3.9% (0.028833 0.0537869	0.88 (0.34–2.31), *p* = 0.802
Adherence to IMS^a^	No	438, 39.7% (36.9–42.6)	85, 60.7% (.52.4–68.4)	353, 36.7% (.33.7–39.7)	2.66 (1.84–3.84), *P*<001
**Block 2: micro level: health care provider**					
Satisfaction with the transplantation team^a^	From 1 to 100	1102, 96.9–8.1 (96.5–97.5)	140, 97.2 ± 7.1 (96.0–98.4)	962, 97.0 ± 8.3 (96.4–97.5)	1.0 (0.98–1.02), *p* = 0.649
Trust in the transplantation team^a^	From 1 to 100	1102, 97.9–61 (97.5–98.2)	140, 96.7–7.9(95.4–98.0)	962, 98.0–5.8 (97.7–98.4)	0.97 (0.94–1.00), *p* = 0.021
**Block 3: meso level: transplant center (characteristics and practice patterns in view of chronic illness management)**					
**Structural characteristics**					
Transplant center region^b^	North/Northeast/Midwest	267, 24.2% (21.7–26.8)	33, 23.6% (17.3–31.3)	234, 24.3% (21.7–27.1)	Reference
	South/Southeast	836, 75.8% (73.2–78.3)	107, 76.4% (68.7–82.7)	729, 75.7% (72.9–78.3)	0.94 (0.56–1.58), *p* = 0.815
Transplant center activity (number of transplants/year in the last 5 years)^b^	1. Low (<50)	422, 38.2% (35,4–41,1)	58, 41.4% (33,6–49,7)	363, 37.7% (34,7–40,8)	Reference
	2. Moderate (50‐150)	398, 36% (33,2–38,9)	39, 27.9% (21,1–35,8)	358, 37.1% (34,2–40,3)	0.67 (0.44–1.02), *p* = 0.065
	3. High (>150)	285, 25.8% (23,3–28,5)	43, 30.7% (23,6– 38,8)	242, 25.1% (22,5–28,1)	1.12 (0.75–1.68), *p* = 0.569
Satisfaction with the comfort of the waiting room ^a^	Yes	472, 42.89% (39.9–45.7)	54, 38.6% (30.9–46.9)	418, 43.5% (40.4–46.6)	0.83 (0.57–1.21), *p* = 0.341
Satisfaction with the cleanliness of the unit ^a^	Yes	210, 19.1% (16.8–21.4)	25, 17.9% (12.3–25.1)	185, 19.2% (16.8–21.8)	0.95 (0.59–1.53), *p* = 0.845
Difficulty accessing the center by public transportation ^a^	Yes	150, 13.6% (12.0–16.0)	18, 13.0% (8.2–19.4)	132, 13.7% (12.0–16.0)	0.95 (0.55–1.63), *p* = 0.854
**Operational characteristics**					
Difficulties in appointment scheduling ^a^	Yes	114, 10.3% (8.7–12.3)	25, 18.0% (12.4–25.3)	89, 9.3% (7.6–11.3)	2.20 (1.35–3.60), *p* = 0.002
Frequency of appointments ^a^	1. Once or more a month	181, 16.4% (14.4–18.9)	32, 22.9% (16.6–30.5)	149, 15.5% (13.3–17.9)	0.62 (0.40–0.96), *p* = 0.035
	2. Every 2 or more per month	922, 83.6% (81.1–85.5)	108, 77.1% (69.4–83.3)	814, 84.5% (82.1–86.7)	0.21 (0.14–0.32), *P*<0.001
Adequacy of number of appointments ^a^	Yes	953, 86.6% (84.4–88.5)	122, 87.8% (81.2–92.3)	831, 86.4% (84.1–88.4)	1.18 (0.68–2.05), *p* = 0.551
Operation of the outpatient clinic ^a^	1. 4 or more times a week	940, 85.2% (83.0–87.2)	117, 83.6% (76.4–89.0)	823, 85.5% (83.1–87.5)	Reference
	2. Up to 3 times a week	163, 14.8% (12.8–17.0)	23, 16.4% (11.2–23.5)	140, 14.5% (12.4–17.0)	1.16 (0.66–2.03), *p* = 0.612
Type of appointment schedule	1. Order of arrival	435, 39.4% (36.5–42.3)	55, 39.3% (31.5–47.6)	380, 39.5% (36.4–42.6)	Reference
	2. Individual schedule	668, 60.6% (58.0–63.5)	85, 60.7% (52.4–68.4)	583, 60.5% (57.4–63.6)	0.87 (0.54–1.41), *p* = 0.584
Follow‐up by the same professionals^a^	Yes	608, 55.1% (52.2–58.1)	75, 53.6% (45.3–61.7)	533, 55.3% (.52.2–58.5)	0.98 (0.62–1.5), *p* = 0.935
If an appointment is missed ^a^	1. Waiting for the next return	180, 16.3% (14.2–18.6)	25, 17.9% (12.3–25.1)	155, 16.1% (14.0–18.5)	Reference
	2. Call absentees	180, 16.3% (14.2–18.6)	25, 17.9% (12.3–25.1)	155, 16.1% (14.0–18.5)	180, 16.3% (14.2–18.6)
Team composition: doctor, nurse, others^a^	Yes	1057, 95.8% (94.5–96.9)	134, 95.7% (90.8–98.1)	923, 95.9% (94.4–97.0)	0.95 (0.34–2.70), *p* = 0.933
Adequate numbers of professionals^a^	Yes	493, 44.9% (42.0–48.0)	65, 47.0% (39.0–55.1)	428, 45,0% (41.5–48.0)	1.11 (0.77–1.60), *p* = 0.577
**Block 4: macro level: health policies**					
Distance from the transplant center^a^	1. Lives in the city of the transplant center	43, 31.1% (28.5–33.9)	49, 35.0% (27.5–43.2)	294, 30.5% (27.7–33.5)	Reference
	2. Does not live in the city of transplant center	760, 68.9% (66.1–71.5)	91, 65.0% (56.7–72.4)	669, 69.5% (66.5–72.3)	0.81 (0.55–1.18), *p* = 0.268
Private health insurance^a^	Yes	258, 23.4% (21.0–26.0)	31, 22.1% (16.0–29.8)	227, 23.6% (21.0–26.4)	0.91 (0.59–1.39), *p* = 0.652

*Note:* Variables highlighted in light grey showed association to be included in multivariate analysis: odds ratio (OR) < 0.75 or > 1.25 and p ≤ 0.25 or *p* < 0.05, independent of the OR value.

Abbreviations: CKD, chronic kidney disease, GFR, glomerular filtration rate; KT, kidney transplantation.

^a^ Variable scored based on the patient's perspective (patient self‐report).

^b^ Variable scored based on centers perspective (transplant center representative).

The prevalence of NApp was 12.7%, as mentioned before [28]. A bivariate descriptive analysis (adherent and non‐adherent) of the multilevel factors related to NApp was performed (Table 1) and 13 variables were selected (9 at the patient level, 1 at the micro level, and 3 at the meso level), by association level, to be included into the multiple model, whose results of the sequential stages are presented in Table 2.

**TABLE 2:** Multivariate analysis of NApp by sequential logistic regression.

Variables^a^, ^b^	Odds ratio	95% confidence interval	p
**Block 1: patient level: non‐modifiable and behavioral variables**			
Age	0.97	0.96–0.99	0.001
Time post‐KT > 5 years	1.86	1.28–2.71	0.001
Use of everolimus	1.58	0.97–2.54	0.062
Nonadherence to IMS	2.55	1.76–3.70	<0.001
**Block 2: patient and micro levels**			
Age	0.97	0.96–0.99	0.001
Time post‐KT > 5 years	1.84	1.26–2.68	0.002
Use of everolimus	1.55	0.96–2.52	0.071
Nad to IMS	2.55	1.75–3.69	<0.001
Team trust scale	0.97	0.95–1.00	0.076
**Final model Block 3: Patient, micro, and meso levels**			
Age	0.97	0.96–0.99	0.001
Time post‐KT > 5 years	2.03	1.38–3.00	<0.001
Nad to IMS	2.41	1.66–3.50	<0.001
Team trust scale	0.98	0.95–1.00	0.079
Monthly frequency of consultations	1.75	1.10–2.77	0.018
Scheduling difficulty	1.91	1.16–3.17	0.011

*Note:* Multivariate analysis by sequential logistic regression: variables selected by the association in the bivariate analysis (*p* value < 0.10 and an effect ≥ 25% by OR) in blocks (1–3) accordingly to the studied levels. To be retained in each block for the next analysis, variables should present an effect ≥ 25% by OR and a *p* value < 0.10.

^a^ Variables included in Block 1, patient level, nonmodifiable and behavioral (9): Sociodemographic factors: age, gender (male), marital status (stable partner); Condition‐related factors: donor type (deceased), time post‐kt (>5 years), use of everolimus, use of azathioprine, nonadherence to immunosuppressives, Block 2, micro level, perceptions of health care team (1): trust in health care team (scale 1–100). Block 3, meso level, opinions/perceptions of patients regarding the center and aspects of clinical practice (3): Structural characteristics: center transplant activity (moderate); Practice patterns: frequency of consultations (monthly), difficulties in scheduling appointments.

^b^ Any macro‐level variables were not included because they did not reach the defined criteria of association.

The final model showed socioeconomic factors (age), treatment‐related factors (KT time >5 years), and behavioral factors (nonadherence to IMS) as independent factors associated with NApp at the patient level. At the micro level (health professional), we found an association with trust in the health team and, at the meso level (health centers), NApp was related to the frequency of consultations and difficulty in scheduling (Table 2). For each year of age, we found a 3% reduction in the likelihood of being less adherent to consultations (OR 0.97, 95% confidence interval [CI]: 0.96–0.99). Meanwhile, patients with more than 5 years post‐KT were twice as likely to be nonadherent to consultations (OR: 2.03; 95% CI: 1.38–3.00), and patients who were not adherent to IMS were almost two and a half fold more likely to have NApp (OR 2.41; 95% CI: 1.66–3.50). Regarding the health team (micro level), for each point increased in the trust scale, there was a 2% reduction in the likelihood of being nonadherent to consultations (OR: 0.98; 95% CI: 0.95–1.00). Regarding the KT center (meso level), patients with frequent consultations (monthly) were 75% more likely to be less adherent to consultations (OR: 1.75; 95% CI: 1.10–2.77), and patients with scheduling difficulties were almost twice as likely to have NApp (OR: 1.91; 95% CI: 1.16–3.17).

## 4 Discussion

This Brazilian multicenter study identified independent multilevel factors associated with NApp after KT. It is the first study to evaluate the association of health system factors (team and service) with missing appointments after KT, and is one of the few studies evaluating NApp after KT [16, 17, 18, 19, 20, 21, 22, 25, 26]. The identified patient factors (age, time post‐KT, and nonadherence to IMS) enable the recognition of patients at risk of NApp, additionally to team and center factors (trust in the team, frequency, and difficulty in scheduling) point to the need for multilevel interventions to target NApp. The strength of this study lies in its multicenter design, involving over a thousand patients from transplant centers with different characteristics, such as transplant activity, geographic regions, and socioeconomic profiles. In addition, a theoretical approach, the ecological model, guided the selection of variables, thereby identifying potential multilevel targets for interventions [24].

Depending on the method used (measurement method and operational definition), the prevalence of NApp varies widely (7.6% to 46.6%) [16, 17, 18, 19, 20, 21, 22]. However, the prevalence in our study [28] was higher than the 7.7% reported in a Turkish study that used the same operational definition [17]. In this sample with a high prevalence of NApp, 12.7% [28], 3 patient‐level variables were included in the final model. As described by others [19, 21, 22], younger patients are more likely to be less adherent to consultations. We found a positive association between longer time after KT (> 5 years) and NApp as reported by Zhao et al. even applying another operational definition of NApp [25]. A qualitative study that assessed patients' perceptions and beliefs and perceived barriers to regularly attending post‐KT appointments reported an increase in self‐confidence with a longer time after KT, with the belief of greater knowledge about their bodies and, therefore, the perception of less need for visits to the KT service.[26] Likewise, longer post‐KT periods are a recognized risk factor for nonadherence to IMS [29, 30, 31, 32, 33]. Similar to our findings, nonadherence to IMS has been previously associated with NApp [19], assessed with varying measurement methods, that is, medication possession rate <80% for nonadherence to IMS and electronic records for NApp. We have previously reported the prevalence and correlates of nonadherence to IMS in this sample [29]. Of particular interest, nonadherence to IMS has psychosocial determinants, such as low self‐efficacy and depression, which may also explain the nonadherence to post‐KT appointments [31, 34, 35]. Meanwhile, NApp is easier to detect compared to nonadherence to IMS, for which there is no consensus on which would be the most accurate diagnostic method for use in clinical practice [36, 37].

This is the first NApp study that reports association with variables beyond the patient‐level [16, 17, 18, 19, 20, 21, 22, 25, 26]. At the micro level, greater trust in the healthcare team was negatively associated with NApp. Although the quality of the team‐patient relationship, measured, for example, by trust in the team, was not reported in relation to post‐KT consultations, in other chronic conditions (HIV infection, diabetes, and systemic lupus erythematosus), it was related to better medication adherence [38, 39, 40, 41]. Additionally, for the first time, patients with more frequent appointments and scheduling difficulties were positively associated with NApp. Although it seems reasonable, these aspects have never been previously evaluated in KT. To ensure appropriate follow‐up after KT, the services are organized to provide consultations at a frequency that allows for the necessary surveillance of immunological and infectious events and management of other morbidities. The system used, however, varies according to local characteristics and resources. Even though KT promotes a better quality of life, patients report post‐KT treatment as a stressor, due to the mandatory use of IMS and constant surveillance of graft function by laboratory tests and consultations [42, 43]. In patients using HIV antiretroviral therapy, interventions with an appointment system monitoring, which included shared decisions about the best time and day for appointments and active surveillance of absences, resulted in a higher frequency of appointments and were also used as indicators of the performance of the service provided by the care units [44].

Identifying multilevel factors allows for the planning of more comprehensive actions to enhance engagement in post‐KT treatment, including attendance at scheduled appointments. To date, no study has reported interventions aimed at reducing NApp in KT patients. The profile of individual and non‐modifiable characteristics (age and post‐KT periods) can be useful to identify patients at risk and thus individualize the proposed treatment. More importantly, efforts to improve the pattern of care, including the behavior of the team professionals and the improvement of the appointment system (adapting to the patient and facilitating access to appointments) and, therefore, the clinical practice of the KT centers, have considerable potential to reduce absences. Recently, the COVID‐19 pandemic has driven major changes in post‐KT care, introducing eHealth technologies as a strategy to enable follow‐up without in‐person appointments. Globally, technologies such as telesupport and teleconsultation have proven effective in enhancing patient engagement with KT centers and preventing complications related to loss of follow‐up [45]. Specifically, this approach could address our findings regarding difficulties with appointment frequency and scheduling when combined with standard care [45, 46]. These changes are in line with the principles of the chronic illness management model, which include: ensuring access and continuity of care; providing opportunities for patients to participate in the care process; integration of the various levels of care and promoting support for self‐management [5]. Improvements in self‐management can be achieved with a better knowledge about the treatment after KT, encouraging active participation in decision‐making and adapting treatment needs to patients' daily routine, thus reducing acute and chronic complications, improving clinical outcomes, and quality of life [5, 47]. There are reports of improvement in clinical outcomes in other populations with chronic diseases, such as diabetes and cardiovascular disease, after the adoption of the chronic illness management model [48]. Although this model has been proposed in the treatment of CKD [47], reports are scarce in KT [15, 49].

### 4.1 Limitations

The cross‐sectional design of the study limits the ability to make causal inferences from the observed associations. The use of a self‐report questionnaire may underestimate NApp when compared to the checking of attendance at appointments in the electronic systems of scheduling/medical records of patients. However, the method used can be applied in clinical practice, can be used at any point in the post‐KT follow‐up, in services without electronic recording systems, and has been used in research after solid organ transplantation [17, 50]. As this is an analysis of secondary data, variables potentially associated with NApp may not have been considered. However, we believe that the multilevel analysis performed can be a starting point for other variables to be included in the future, such as those that assess the quality of health care, further aspects of the scheduling system (virtual), and at the macro level (health system and policies, e.g., payment for consultations). The data were collected before the COVID‐19 pandemic, before the widespread adoption of eHealth technologies in post‐KT care. Accordingly, future studies should focus on how telemedicine will continue to be integrated into hybrid care models, its impact on patient outcomes, and the necessity and application of regulatory standards [45, 46].

## 5 Conclusions

This study was the first to evaluate the multilevel factors associated with NApp after KT. The results showed the association with variables at the micro and meso levels and therefore beyond the patient level, suggesting the need for a care model that focuses on the quality of the team‐patient relationship as well as on the dynamics in the clinical practices of transplant centers to reduce the behavior of NApp and its undesirable outcomes.

## Author Contributions

Roberta Lopes Karlburger wrote the first version of the paper. Helady Sanders‐Pinheiro, Sabina De Geest, and Fernando Antônio Basile Colugnati participated in the research design. Fernando Antônio Basile Colugnati, João Henrique Sendrete de Pinho, and Kris Denhaerynck. performed the data analysis. All the authors participated in data collection, revision, and final approval of the manuscript.

## Conflicts of Interest

The authors declare no conflicts of interest.

## The ADHERE BRAZIL Study Team Members

**Alvaro Pacheco‐Silva:** Hospital Israelita Albert Einstein, São Paulo, Brazil; **Carlos G.W.C. Marmanillo:** Hospital Angelina Caron, Curitiba, Brazil; **Deise B.M. Carvalho:** Hospital São Francisco de Assis da Providência de Deus, Rio de Janeiro, Brazil; **Elias David‐Neto:** Hospital das Clínicas ‐ University of Sao Paulo School of Medicine, São Paulo, Brazil; **Giuseppe C. Gatto:** Hospital Universitário de Brasília, Brasília, Brazil; **Gustavo F. Ferreira:** Santa Casa de Juiz de Fora, Juiz de Fora, Brazil; **Luciane M. Deboni:** Fundação Prorim ‐ Hospital Municipal de São José, Joinville, Brazil; **Marilda Mazzali:** State University of Campinas, Campinas, Brazil; **Mário Abbud‐Filho:** Hospital de Base, São José Rio Preto, Brazil; **Maurício G. Pereira:** Federal University of Rio Grande do Norte‐Hospital Onofre Lopes, Natal, Brazil; **Paula F.C.B.C. Fernandes:** Federal University of Ceará, Fortaleza, Brazil; **Pedro A.M. Souza:** Santa Casa de Misericórdia de Belo Horizonte, Belo Horizonte, Brazil; **Rafael F. Maciel:** Hospital Antonio Targino, Campina Grande, Brazil; **Roberto C. Manfro:** Federal University of Rio Grande do Sul‐Hospital das Clínicas de Porto Alegre, Porto Alegre, Brazil; **Sergio Wyton:** Hospital São João de Deus, Divinópolis, Brazil; **Silvia R. Cruz:** Federal University of Para‐Hospital Ofir Loyola, Belém, Brazil; **Teresa C.A. Ferreira:** Federal University of Maranhão ‐ Hospital Universitário do Maranhão, São Luiz, Brazil; **Valter D. Garcia:** Santa Casa de Porto Alegre, Porto Alegre, Brazil.

## Supporting information




**Supporting Data 1:** Multilevel correlates of nonadherence routine outpatient appointments collected in the ADHERE BRAZIL study.

## Data Availability

The data on which the analyses are based are available on the Open Science Framework website at https://osf.io/zg97b/ and https://osf.io/qhmdg/, upon the authors´ request.
