# Supplementary material for: Associated Factors to Nonadherence to Routine Appointments after Kidney Transplantation: The ADHERE Brazil Study
Source: Clin Transplant. 2025 Oct 7;39(10):e70339. doi: 10.1111/ctr.70339 (PMC12503078; doi:10.1111/ctr.70339)
Supplement: Supplementary file 1 — Supporting Data 1: Multilevel correlates of nonadherence routine outpatient appointments collected in the ADHERE BRAZIL study. [file CTR-39-e70339-s001.docx]

**Supplementary data**

**Supplementary data 1.** Multilevel correlates of nonadherence routine outpatient appointments collected in the ADHERE BRAZIL study.

| **Variable** | **Instrument** | - **Assessment method** - **Conceptual definition (if needed)** - **Number of items** - **Recall period** - **Response options** - **Scoring** | **Comment, reference** |
| --- | --- | --- | --- |
| **Patient Level** | | | |
| **SOCIODEMOGRAPHIC FACTORS** | | | |
| Age | Investigator-developed self-report questionnaire based on previous transplant research | - Structured patient interview - 01 item - Years - Continuous variable | [1-6] |
| Sex | Investigator-developed self-report questionnaire based on previous transplant research | - Structured patient interview - 01 item - Male/female - Dichotomous variable | [1-8] |
| Education level | Investigator-developed self-report questionnaire based on previous transplant research | - Structured patient interview - 01 item - 4 categories: Illiterate (0-4 years), Elementary school (4-8 years), High school (> 8 to 11 years), College (<11 years) - Ordinal variable | [1,2,4,5,8] |
| Race | Investigator-developed self-report questionnaire based on previous transplant research | - Structured patient interview - 1 item - Caucasian/white vs. non-Caucasian (other ethnicities: Mixed, African-American, Asiatic, Indian) - Dichotomous variable | [1,3,5] |
| Marital status | Investigator-developed self-report questionnaire based on previous transplant research | - Structured patient interview - 1 item - Steady partner (married, living together) or without steady partner (single or divorced/separated or widowed) - Dichotomous variable | [2-4,8] |
| Working status | Investigator-developed self-report questionnaire based on previous transplant research | - Structured patient interview - 1 item - Actively employed [being (self) employed] vs. not actively employed (unemployed, unable to work/receiving social benefits, retired, housewife, student) - Dichotomous variable | [4,8] |
| Familiar income | Investigator-developed self-report questionnaire based on previous transplant research | - Structured patient interview - 1 item - 4 categories: Up to 1 reference wage/>1 to 3 wages/>3 to 5 wages/more than 5 wages - Ordinal variable | Based on Brazilian reference wage/month = approximately U$ 248.28.  [1,4,8] |
| Religion | Investigator-developed self-report questionnaire based on previous transplant research | - Structured patient interview - 1 item - 5 categories: Catholic/Protestant/Spiritual/No religion/Other - Categorical variable | [9] |
| **CLINICAL FACTORS** | | | |
| Chronic kidney disease aetiology | Investigator-developed self-report questionnaire based on previous transplant research | - Structured form for medical record information extraction - 1 item - 5 categories: Chronic glomerulonephritis/Undetermined/Hypertensive Nephropathy/Diabetic Nephropathy/Polycystic Kidney Disease/Other - Categorical variable | [8] |
| Pre-KT treatment modality | Investigator-developed self-report questionnaire based on previous transplant research | - Structured form for medical record information extraction - 1 item - 3 categories: Hemodialysis/Peritoneal dialysis/Preemptive - Categorical variable | [3,6,8] |
| Acute rejection | Investigator-developed self-report questionnaire based on previous transplant research | - Structured form for medical record information extraction - 1 item - Yes/No - Dichotomous variable | [3,5] |
| Creatinine | Investigator-developed self-report questionnaire based on previous transplant research | - Structured form for medical record information extraction - 1 item - Last value recorded in medical file at the time of enrolment - Continuous variable | [6] |
| Estimated Glomerular Filtration Rate (GFR) | Estimated by CKD-EPI equation.[10] | - Structured form for medical record information extraction - 1 item - Calculated based on the last value of creatinine registered in the medical file at the time of enrolment - Continuous variable | [6] |
| Chronic kidney disease, categories | Based on Glomerular filtration rate estimated by CKD-EPI equation.[10] | - Structured form for medical record information extraction - 1 item - 5 categories: 1 (GFR >90)/2 (GFR 90-60)/3A (GFR 59-45)/3B (GFR 44-29)/4 (GFR 30-15)/5 (GFR <15) - Ordinal variable | [9] |
| Number of hospitalizations KT, categories | Investigator-developed self-report questionnaire based on previous transplant research | - Structured form for medical record information extraction - 1 item - 4 categories: None/One/Two to three/More than three - Ordinal variable | [5] |
| **TREATMENT-RELATED FACTORS** | | | |
| Time since transplantation, categories | Investigator-developed self-report questionnaire based on previous transplant research | - Structured form for medical record information - 1 item - Up to 5 years vs. more than 5 years - Dichotomous variable | [4,8] |
| Type of donor | Investigator-developed self-report questionnaire based on previous transplant research | - Structured form for medical record information extraction - 1 item - Living vs. deceased donor - Dichotomous variable | [3-8] |
| Immunosuppressives | Investigator-developed self-report questionnaire based on previous transplant research | - Structured form for medical record information extraction - 8 items - Each item assessed as a dichotomous yes/no score (actual), recorded in the medical file at the time of enrolment: prednisone, tacrolimus, sodium mycophenolate, azathioprine, cyclosporine, everolimus, mycophenolate mofetil, sirolimus - Dichotomous variable | [3,5,8] |
| Number of daily doses of immunosuppressives | Investigator-developed self-report questionnaire based on previous transplant research | - Structured form for medical record information extraction - 1 item - Maximum number of daily doses of immunosuppressives taken/day (once/two times a day vs. or 3 or more times a day) - Dichotomous variable | [9] |
| **BEHAVIOURAL FACTORS** | | | |
| Nonadherence to appointment keeping |  | - Structured patient interview - 1 item - Last 5 appointments recall period - Nonadherent = missed ≥ 1 appointment - Dichotomous variable | [11] |
| Nonadherence to physical activity recommendations | Adapted from the Brief Physical Activity Assessment tool [12] | - Structured patient interview - 2 items - Questions: Do you perform any physical activity, yes/no. If yes, # min/week - Sufficiently active: ≥ 150 min/week of at least moderate physical activity. Nonadherent = not being sufficiently active - Dichotomous variable | [9,13] |
| Smoking status | Investigator-developed self-report questionnaire based on previous research | - Structured patient interview - 1 item - 1-year recall period - 4-point Likert scale; 1 = yes to 4 = no, I never smoked. - Non-adherence= patients who reported to currently smoke - Dichotomous variable | [5,7] |
| Nonadherence to immunosuppressives | Basel Assessment of Adherence with Immunosuppressive Medication Scale - BAASIS | - Structured patient interview - 4 items implementation phase - Nonadherent: positive response to 1 of the 4 questions of the BAASIS - Dichotomous variable | [5,9,14] |
| **Micro level: Healthcare provider** | | | |
| Patient´s satisfaction with the transplant team | VAS scale | - Structured patient interview - 1 item - Current situation - Ranging from 0 = not satisfied to 100 = very satisfied - Continuous variable | [9,15] |
| Patient´s trust in the transplant team | VAS scale | - Structured patient interview - 1 item - Current situation - Ranging from 0 = not satisfied to 100 = very satisfied - Continuous variable | [9,15] |
| **Meso level: transplant centre (characteristics and practice patterns in view of chronic illness management)** | | | |
| **STRUCTURAL CHARACTERISTICS** | | | |
| Transplant centre ADHERE region | Investigator-developed self-report questionnaire based on previous research | - Transplant director questionnaire - 1 item - Current situation - 2 categories (Brazilian geographical region according to number of transplants): North/Northeast/Central West vs. Southeast/South - Categorical variable | [9] |
| Transplant centre activity | Investigator-developed self-report questionnaire based on previous research | - Transplant director questionnaire - 1 item - Current situation - 3 categories (number of transplants/year in the last 5 years); Low (<50)/Moderate (50-150)/High (>150) - Ordinal variable | [9,11] |
| Satisfaction with waiting room structure | Investigator-developed based on previous research | - Structured patient interview - 1 item - Current situation - Yes vs. no - Dichotomous variable | [9,16,17] |
| Satisfaction with the cleanliness of the unit | Investigator-developed based on previous research | - Structured patient interview - 1 item - Current situation - Yes vs. no - Dichotomous variable | [9,16,17] |
| Difficulties in accessing the centre by public transportation | Investigator-developed based on previous research | - Structured patient interview - 1 item - Current situation - Yes vs. no - Dichotomous variable | [9,16,17] |
| **PRACTICE PATTERNS** | | | |
| Difficulties in scheduling appointments | Investigator-developed based on previous research | - Structured patient interview - 1 item - Current situation - Yes vs. no - Dichotomous variable | [9,16,17] |
| Frequency of appointments | Investigator-developed based on previous research | - Structured patient interview - 1 item - Last 6 months - 2 categories: Once or more a month vs. every 2 or more per month - Categorical variable | [9,16,17] |
| Adequacy of number of appointments | Investigator-developed based on previous research | - Structured patient interview - 1 item - Current situation - Yes vs. no - Dichotomous variable | [9,16,17] |
| Operation of the outpatient clinic | Investigator-developed based on previous research | - Transplant director questionnaire - 1 item - Current - 2 categories: 4 or more times a week vs. up to 3 times a week - Categorical variable | [9,16,17] |
| Type of appointment schedule | Investigator-developed based on previous research | - Transplant director questionnaire - 1 item - Current situation - 2 categories Order of arrival vs. individual schedule - Categorical variable | [9,16,17] |
| Patients followed up by the same healthcare professional | Investigator-developed based on previous transplant research | - Transplant director questionnaire - 1 item - Current situation - Yes vs. no - Dichotomous variable | [9,11,18,19] |
| If an appointment is missed | Investigator-developed based on previous transplant research | - Transplant director questionnaire - 1 item - Current situation - 2 categories: Waiting for the next return vs. call absentees - Categorical variable | [9] |
| Multiprofessional team | Investigator-developed based on previous research | - Transplant director questionnaire - 1 item - Current situation - Yes (Doctor+ nurse + other health professional) vs. no - Dichotomous variable | [9,19,20] |
| Adequacy of the number of professionals | Investigator-developed based on previous transplant research | - Structured patient interview - 1 item - Current situation - Yes vs. no - Dichotomous variable | [9,19,20] |
| **Macro level: health policies** | | | |
| Distance from the transplant centre | Investigator-developed based on previous transplant research | - Structured patient interview - 1 item - Current situation - 2 categories: Lives vs not lives in the city of the transplant centre - Dichotomous variable | [2,9] |
| Private health insurance | Investigator-developed based on previous research | - Structured patient interview - 1 item - Current situation - Yes vs. no - Dichotomous variable | [9] |

KT, kidney transplantation

**References**

1. Dew MA, DiMartini AF, De Vito Dabbs A et al. Rates and risk factors for nonadherence to the medical regimen after adult solid organ transplantation. Transplantation 2007; 83: 858–873. https://doi.org/10.1097/01.tp.0000258599.65257.a6

2. Kobus G, Małyszko J, Małyszko JS, Puza E, Bachórzewska-Gajewska H, Myśliwiec M. Compliance with lifestyle recommendations in kidney allograft recipients. Transplant Proc 2011; 43: 2930–2934. https://doi.org/10.1016/j.transproceed.2011.08.031

3. Taber DJ, Fleming JN, Fominaya CE et al. The impact of health care appointment non-adherence on graft outcomes in kidney transplantation. Am J Nephrol 2017; 45: 91–98. https://doi.org/10.1159/00045355427

4. Kenawy AS, Gheith O, Al-Otaibi T et al. Medication compliance and lifestyle adherence in renal transplant recipients in Kuwait. Patient Prefer Adherence 2019; 13: 1477–1486. https://doi.org/10.2147/PPA.S209212

5. Mohamed M, Soliman K, Pullalarevu R et al. Non-adherence to appointments is a strong predictor of medication non-adherence and outcomes in kidney transplant recipients. Am J Med Sci 2021; 362: 381–386. https://doi.org/10.1016/j.amjms.2021.05.011

6. Yamanaga S, Hidaka Y, Kawabata C et al. Appointment nonadherence and graft outcomes in living donor kidney transplantation. Transplant Proc 2023; 55: 748–751. https://doi.org/10.1016/j.transproceed.2023.03.003

7. Yavuz A, Tuncer M, Erdoğan O, et al. Is there any effect of compliance on clinical parameters of renal transplant recipients? Transplant Proc. 2004;36(1):120-121. https://doi.org/10.1016/j.transproceed.2003.11.052

8. Zhao L, Yan J, Yang GL et al. A Study on Adherence to Follow-up, Quality of Life, and Associated Factors Among Renal Transplant Recipients in China. Transplant Proc 2017;49:1285-1290. doi: 10.1016/j.transproceed.2017.03.086.

9. Sanders-Pinheiro H, Colugnati FAB, Marsicano EO, et al. Prevalence and correlates of non-adherence to immunosuppressants and to health behaviours in patients after kidney transplantation in Brazil - the ADHERE BRAZIL multicentre study: a cross-sectional study protocol. BMC Nephrol 2018; 19: 41. https://doi.org/10.1186/s12882-018-0840-6

10. Delgado C, Baweja M, Crews DC et al A Unifying Approach for GFR Estimation: Recommendations of the NKF-ASN Task Force on Reassessing the Inclusion of Race in Diagnosing Kidney Disease. Am J Kidney Dis. 2022;79:268-288.e1. doi: 10.1053/j.ajkd.2021.08.003.

11. Berben L, Denhaerynck K, Dobbels F et al. BRIGHT Study Consortium. Building research initiative group: chronic illness management and adherence in transplantation (BRIGHT) study: study protocol. J Adv Nurs 2015; 71: 642–654. https://doi.org/10.1111/jan.12519

12. Marshall AL, Smith BJ, Bauman AE, et al. Reliability and validity of a brief physical activity assessment for use by family doctors. Br J Sports Med. 2005;39(5):294-297.

13. World Health Organization. Global Recommendations on Physical Activity for Health. Geneva: WHO; 2010.

14. Sanders-Pinheiro H, Colugnati FAB, Denhaerynck K et al. Multilevel correlates of immunosuppressive nonadherence in kidney transplant patients: the multicenter ADHERE BRAZIL study. Transplantation 2021; 105: 255–266. https://doi.org/10.1097/TP.0000000000003214

15. Voutilainen A, Pitkaaho T, Kvist T, et al. How to ask about patient satisfaction? The visual analogue scale is less vulnerable to confounding factors and ceiling effect than a symmetric likert scale. *J Adv Nurs.* 2016;72(4):946-957.

16. Melchior R, Nemes MIB, Basso CR, et al. Evaluation of the organizational structure of HIV/AIDS outpatient care in Brazil. *Rev Saude Publica.* 2006;40(1):143-151.

17. BRASIL. *Qualiaids - Avaliação e Monitoramento da Qualidade e Recomendações de Boas Práticas da Assistência Ambulatorial em AIDS no SUS.* Brasil: Ministério da Saúde; 2008.

18. Berben L, Russell CL, Engberg S, et al. Development, content validity and inter-rater reliability testing of the chronic illness management implementation-building research initiative group: Chronic illness management and adherence in transplantation: An instrument to assess the level of chronic illness management implemented in solid organ transplant programmes. *Int J Care Coord.* 2014;17(1-2):59-71.

19. World Health Organization. Preparing a health care workforce for the 21st century. 2005; http://www.who.int/chp/knowledge/publications/workforce_report.pdf. Accessed June 3, 2018.

20. Bissonnette J, Woodend K, Davies B, et al. Evaluation of a collaborative chronic care approach to improve outcomes in kidney transplant recipients. *Clin Transplant.* 2013;27(2):232-238.
